# Establishing Proof-of-Concept for the Feasibility and Implementation of a Point-of-Sale Firearm Safety Counseling Program: A Qualitative Case Study

**DOI:** 10.1177/15248399251323898

**Published:** 2025-03-24

**Authors:** Amanda Koong, Mike Henson-Garcia, Lauren Malthaner, Katelyn Jetelina, Belinda Hernandez, Sandra McKay

**Affiliations:** 1 McGovern Medical School at UTHealth Houston, Houston, TX, USA; 2 UTHealth School of Public Health, Houston, Texas, USA; 3 Yale School of Public Health, New Haven, CT, USA

**Keywords:** community-based interventions, point-of-sale interventions, stakeholder engagement, firearms, safe firearm storage

## Abstract

Safe firearm storage is critical for injury prevention, yet there is limited evidence surrounding effective interventions to promote storage behaviors among owners. One potential avenue for intervention is at the point-of-sale, a concept well-proven in the nutrition promotion and tobacco cessation space. This study explored firearm retailer attitudes toward a firearm storage intervention delivered at the point of sale. Seventeen in-depth interviews were conducted with firearm retailers from a suburban gun shop in Fort Bend, USA. This semi-structured interview assessed firearm retailer perceptions of the proposed point-of-sale intervention. Interviews were transcribed and independently coded to identify themes that emerged from participants’ statements. Data were organized and analyzed using an inductive–deductive hybrid approach. Most retailers expressed high acceptability and appropriateness of implementing interventions at the point of sale. Six key themes were identified: (a) retailers viewed point-of-sale safe storage programs as acceptable; (b) the appropriateness of interventions varied depending on customer group; (c) specific program design considerations were important for successful implementation; (d) retailer self-efficacy differed across retailers; (e) barriers and (f) facilitators to consider for successful program implementation. Results indicate general favorability toward point-of-sale programs given supportive infrastructures. Interventions delivered through trusted relationships in firearm shops represent a promising approach to promoting safer storage. However, important nuances and barriers were also identified.

## Introduction

The most recent data from the Centers for Disease Control and Prevention has determined that firearm-related suicide mortality rate reached an all-time high of 8.1 per 100,000 in 2022—representing a major public health issue in the United States (Baumgaertner, 2023). Time and time again, safe storage of firearms has been implicated as an important and modifiable determinant of firearm-related suicide; yet, it has been estimated that over 50% of gun owners in the United States store their firearms unlocked and or loaded (Rowhani-Rahbar et al., 2016). Increasing proper firearm storage could prevent a substantial proportion of suicides, given previous empirical evidence suggesting that storing firearms in a locked and unloaded manner is protective against firearm-related suicides (Grossman et al., 2005; Monuteaux et al., 2019).

In addition, suicide attempt by firearm has a high case fatality rate (90%)—accounting for nearly 50.6% of suicide deaths annually, despite firearms only being implicated in ~4.8% of suicide attempts (Bandel et al., 2023; Conner et al., 2019; Spicer & Miller, 2000; Wang et al., 2020). In addition, a nationally representative study of next-of-kin to “all-cause” suicide decedents concluded that decedents living in households without safe storage practices were more likely to die by *firearm*-related suicide, as opposed to other suicide mechanisms than those who lived in households with safe storage practices (Rowhani-Rahbar et al., 2016; Shenassa et al., 2004). Altogether, the evidence to date supports safe storage (i.e., keeping a firearm locked and unloaded) as a key mitigating practice in the prevention of impulsive suicides by reducing easily accessible and extremely lethal means (i.e., firearms).

Currently, there exists a need for the development, implementation, evaluation, and dissemination of evidence-based firearm safe storage interventions—particularly programs that are culturally sensitive to the perspectives of the firearm-owning community (Betz et al., 2022; Cunningham et al., 2019). In response, there have been specific calls to engage firearm retailers in suicide prevention activities (Polzer et al., 2021) as well as a growing focus on gun establishments as potential settings for public health interventions. Termed “gun shop projects,” these community-based suicide prevention initiatives are underway in over 20 states, and the results of these interventions will likely be reported over the next few years (Barber et al., 2017). Interviews with leaders of gun shop projects indicate that the point of sale was an important juncture for disseminating prevention efforts (Polzer et al., 2021).

Point-of-sale interventions, particularly designed in the context of tobacco cessation and obesity prevention, have been demonstrated as a highly effective approach to spurring behavior change. For instance, a systematic review conducted by Robertson et al. (2015) determined that interventions implemented at the point of sale via tobacco display bans increased the odds of cigarette smoking cessation/reduction via e-cigarettes by 1.46 (Robertson et al., 2015). Similarly, a systematic review by Liberato et al. (2014) indicated that nutrition interventions in the form of monetary incentives at the point of sale increased the purchasing of healthier foods (Liberato et al., 2014). Within the context of firearm safety, point-of-sale interventions have not yet been developed, although they have been suggested as a promising strategy for the prevention of firearm injury (Bandel et al., 2023). It is essential to acknowledge that point-of-sale interventions for tobacco cessation and obesity prevention exhibit certain distinctions from the proposed firearm safety intervention. Specifically, the latter is intended to influence delayed behaviors related to the secure storage of firearms within the home, whereas tobacco and obesity interventions predominantly aim to modify behaviors at the point of purchase. Nonetheless, it is important to consider that a likely outcome of firearm storage counseling interventions, delivered at the point-of-sale, will be an increase in the acquisition of safe storage equipment, which parallels the objectives of point-of-sale interventions in these other domains.

Numerous studies have underscored firearm owners’ preferences to receive firearm safety information from trusted messengers, which are typically those individuals possessing practical experience in handling firearms (e.g., firearm retailers, veterans, etc.) (Anestis et al., 2022; Ewell-Foster et al., 2024). Concurrently, previous research has indicated that thoughtful, culturally sensitive firearm safety counseling delivered by pediatricians during well-child visits is effective in promoting safe storage behavior (Kaufman et al., 2023). Coupling the effectiveness of counseling in pediatric clinics with a preference for firearm safety information from trusted messengers, it is plausible to investigate whether firearm safety interventions should be adapted for contexts that are more culturally congruent with firearm owners, such as gun shops and other gun establishments. However, to date, no peer-reviewed evidence has examined the acceptability, appropriateness, and feasibility of adapting these anticipatory guidance programs into these novel settings.

Thus, the objective of this present study was to explore retail staff perceptions toward a proposed interpersonal communication intervention delivered at the point-of-sale, which aims to promote firearm safe storage practices among customers. In addition, this study set out to identify potential barriers and facilitators related to the implementation of these anticipatory guidance programs within these unique settings.

## Method

### Research Team, Reflexivity, and Positionality Statement

The research team consisted of several members each with diverse perspectives and experiences relevant to the topic of study. This included AK, a second-year medical student with a background in prevention research. AK is not a firearm owner. MHG is a fourth-year doctoral candidate studying health promotion and epidemiology. MHG is trained in qualitative methodology and has developed expertise in areas including intervention development, implementation science, health communication, and behavioral sciences. As a non-firearm owner who has lost friends and family to firearm injury, MHG brings a valuable perspective to the team. LM, a fourth-year doctoral candidate in epidemiology and biostatistics with previous training in social and behavioral sciences, provided expertise in injury and violence epidemiology. LM does not own firearms but has also been indirectly impacted by firearms and firearm injury in her family. KJ is an epidemiologist with expertise in injury and violence prevention and is a firearm owner. BH is a behavioral and implementation scientist with expertise in developing, implementing, and evaluating interventions to prevent risky behaviors and violence among youth and young adults. BH is also a firearm owner. SM is a pediatrician with expertise in firearm injury advocacy and also owns a firearm.

While shaped by our backgrounds, the ultimate goal in conducting and reporting this research is to foreground the voices, experiences, and norms of the communities we study over our individual and collective assumptions. This reflexive posture enabled our team to critically evaluate our methodology and findings to produce knowledge with nuance.

### Study Design, Procedure, and Participants

In May 2022, the principal investigator (SM) visited the study site several times to inform participants of the study, answer any questions, and enroll them for participation, including informed consent. Over 2 months, site visits were conducted on varied days of the week and times of the day to capture the diversity of the staff. Between June through August 2022, semi-structured interviews were conducted with 17 firearm retail associates (100% of current store employees). While these firearm retail associates were all employed at the same medium-sized firearm shop in the suburbs of Fort Bend, Texas, their responsibilities encompassed a broad range, including retail associates, armorers, firearm safety instructors, range officers, member services managers, and upper management. This store primarily serves the surrounding county. Further population demographics are detailed in Table 1 (“Fort Bend County, Texas—Census Bureau Profile,” n.d.; “Fort Bend County, TX Data USA,” n.d.).

**Table 1:** Fort Bend, Texas Demographic Table (Population Size = 83,000)

*Fort bend, Texas*	
Age (median)	36.9
Sex	
Female	50.80%
Male	49.20%
Income	
Median	$105,205
Less than $10,000	3.60%
$10,000 to $24,999	5.61%
$25,000 to $49,999	12.90%
$50,000 to $99,999	25.70%
$100,000 to $149,999	19.70%
$150,000 to $199,999	13.80%
$200,000 or more	19.00%
Education (25 years +)	
High school or equivalent	16.2%
Some college, no degree	17.8%
Associate’s degree	7.5%
Bachelor’s degree	31.5%
Graduate or professional degree	18.8%
Race	
White Non-Hispanic	30.3%
Black or African American Non-Hispanic	20.0%
Asian Non-Hispanic	21.1%
White Hispanic	10.7%
Multiracial Hispanic	8.03%
Other Hispanic	5.6%
Multiracial Non-Hispanic	3.1%
Voting record (2020 presidential election)	
Donald Trump, Mike Pence	44.01%
Joe Biden, Kamala Harris	54.57%

This location was selected for a few reasons. First, its capacity as a medium-sized community-oriented store reflected the type of retailer we aim to design this intervention toward. Community-oriented stores are critical in leveraging the preexisting trust and dialogue they have with their customers. In addition, the store owner was supportive of firearm safety initiatives and thus, had mutually aligned interests with our team members.

Interviews were conducted via WebEx virtual conferencing with MHG. All interviews were video and audio recorded and professionally transcribed. The length of interviews ranged between 40 to 90 minutes. Transcripts were de-identified before data analysis to protect participant confidentiality. In appreciation for their time, all participants each received a $50 Amazon gift card upon completing the interview. The Committee for the Protection of Human Subjects at the University of Texas Houston Health Science Center reviewed and approved this study (IRB# HSC-MS-22-0002).

### Theoretical Frameworks and Intervention Concepts

The study team developed a semi-structured interview guide (Supplemental Appendix), with 25 questions and associated probing questions to better understand perceptions surrounding the implementation of firearm safety interventions at the point of firearm sale. The interview guide began by asking about the subject’s current occupational responsibility and typical customer interactions. After discussing their attitudes and beliefs surrounding firearm safety in society as well as the effectiveness of firearm safety devices, the interviewer segued into a proposed firearm safety intervention at the point-of-sale. Regarding the potential intervention, participants were only provided the following details:

In our program, we would train firearm retailers to provide information and counsel individuals around three different topics: (1) proper firearm storage, (2) keeping firearms away from children, and (3) keeping firearms away from those experiencing suicidal ideation. Keep this information in mind as we go through the next series of questions

Guided by core concepts from the implementation outcomes framework (Proctor et al., 2011), this second segment of the interview captured information about the retailers’ perceptions concerning the acceptability and appropriateness of the proposed intervention. Acceptability is the degree to which implementation stakeholders view an intervention as satisfactory (Proctor et al., 2011). Appropriateness is defined as the perceived fit of an intervention within a specific setting (Proctor et al., 2011). In addition, the interview guide included questions surrounding implementation self-efficacy, implementation barriers, and implementation facilitators.

### Data Analysis

Coding was independently conducted by MHG and LM, to categorize and organize the transcript data. The two coders employed a hybrid structural and open coding process. Structural coding categorized interview excerpts according to the corresponding questions in the interview guide. Open coding involved an inductive analysis of the data to identify concepts and meanings that emerged directly from the participants’ statements. This combination allowed the coders to systematically organize the data while also capturing unanticipated themes grounded in the respondents’ worldviews. MHG and LM met regularly to discuss the evolving codebook and ensure consistent interpretation and application of codes. Intercoder percent agreement, calculated by NVivo, ranged from 84% to 100%, indicating strong consistency in the descriptive codes applied across the transcripts.

In the second coding cycle, pattern coding was used to cluster the initial codes into broader themes and explore connections between them. The research team employed an inductive–deductive hybrid approach to identify emergent themes. All data were organized and analyzed in NVivo version 12.

## Results

Overall, seventeen participants completed the study. The participants were predominantly male (*n* = 16/17), and most identified as White (n = 13), followed by Black or African American (n = 3), and Latino/Hispanic (n = 1). Their job roles were diverse and included retail associates, armorers, firearm safety instructors, range officers, member services managers, and upper management. The diversity of perspectives and experiences among the staff provided insight into the inner workings of the firearm shop from different vantage points, allowing for a nuanced understanding of multi-level perspectives regarding the rollout of firearm safety counseling interventions in retail and range environments. Six unique themes emerged from our analysis. Table 2 demonstrates exemplar quotes related to each theme.

**Table 2:** Exemplar Quotes

*Theme*	*Exemplar quotes*
Acceptability	“I think having the system is fantastic. Having the people that are monitoring it and managing it and deploying it is fantastic. But if I, as a salesperson cannot buy into it, cannot help explain it and can’t help point customers to the proper items that they would need, then I think I’m doing the customer an injustice and the program an injustice.” **(Participant 12, Director)** “I think that’s a really good program to instill for sure.” **(Participant 5, Retail Associate and Range Officer)** “So it would just be kind of a good thing to plant in people’s minds, whether they’re thinking about it or not, because it’s just something good to be aware of after purchasing a gun” **(Participant 4, Retail Associate)** “I don’t think there would be anybody who would be opposed.” **(Participant 10, Retail Associate and Armorer)** “I would love to have an opportunity to find out new flaws, these new understandings, get into the more mental psyche, learn more information about warning signs, being able to interact with people better and make them feel more comfortable while being safe. Absolutely. . . you can never obtain enough knowledge or enough safety information.” **(Participant 8, Instructor and Range Director)**
Appropriateness	“Some customers would find it. . . informative and appreciate the information. . . The only time you may come across someone that don’t agree with it, someone that, like I said, says, “I’ve been shooting since I was four years old. I know everything. I know what I’m doing.” But for the most part, most people are very appreciative of the information. They’ll take it in. They’ll learn.” **(Participant 11, Manager)** “There would just maybe be a few that might feel like, "Oh, this is stuff that I already know, so I don’t feel obligated to listen to this, or read this," or whatever. But I would say the very large majority of our customers would probably appreciate that, especially if they’re like first-time gun buyers, or if they’re not very experienced.” **(Participant 4, Retail Associate)** “For newer gun owners, for first-time gun owners, yes, definitely . . . Now, you say it for the older crowd, for people who have known firearms for years and years. . .They say, "Hey, I don’t need you telling me this. I’ve got a thousand guns already. I know how to keep my stuff safe.” **(Participant 1, Manager)**
Adaptation	“So not every store is the same. . .. which is why I’m kind of like advocating for your program being on maybe a tablet, because not every store can have the capacity to have physical presence.” **(Participant 15, Retail Associate)** “And so, in a place like ours I think this concept of this program is very—would be very effective. . .” **(Participant 14, Executive Leader)** Now, I will say that is not the standard across the board . . . It depends on where you go. . . It’s not that something like that couldn’t be implemented, but how likely would it be to have that implemented at the same level across the board?” **(Participant 1, Manager)**
Implementation self-efficacy	“Me personally, I’m very confident in it. Like I said, I’ve been in this industry for a few years now, quite a few years.” **(Participant 1, Manager)** “It’s kind of part of our job. I mean, it is part of our job to do that . . . I think there’s nobody better than the person you’re buying the gun from." **(Participant 2, Manager)** “I’d be open to it. Me personally, I’d be open to it. That’s for the mere fact of how many people I know that’s committed suicide. I know someone that was really close to me that spoke about doing it. I was able to talk her down. And coming through the experience myself of attempting it. I said I’d never do it again. But yeah, I would feel comfortable taking courses and learning, but it’s still a touchy subject for me, too.” **(Participant 11, Manager)** “No. I would say not. I wouldn’t too much want to elaborate on that. . .I’m not going to sell you a firearm and then at the same time start talking to you about suicide issues. No.” **(Participant 13, Range Safety Officer)** “I don’t think I’d be qualified . . . I don’t think [customers would] see that as medical help or a shrink or anything like that. But maybe pointing them in the right direction, maybe that would help. I don’t know. **(Participant 7, Retail Associate)**
Implementation barriers	“Now, making sure everybody does that every single time, that’s a little bit more of a challenge. . . I guess the biggest hurdle would still be the standardization.” **(Participant 10, Retail Associate and Armorer)** “Like I said, I mean, you can have a policy like that, but how likely will it be to have it consistently implemented from employee to employee, to customer to customer.” **(Participant 1, Manager)** “So I think that would be important because there’s, again, we’re talking about retail jobs. There’s high turnover in retail jobs.” **(Participant 12, Director)** “But I can’t just counsel everyone. I don’t know if they’re mentally stable or not, so I don’t want to waste my time or anybody else’s . . . it’s more responsibility. . . We already do a lot of work and adding more training for, I don’t know, I guess for the same pay, I don’t think it would be super appealing to sit someone down to talk about it. I don’t think it would be worth it to them” **(Participant 7, Retail Associate)** “A lot of times some of the stores are understaffed as well, so that also would reduce the amount of influx of customers that we can help at a time.” **(Participant 15, Retail Associate)** “So if we’ve got a line growing because we’re having three, 4-min conversations, it becomes—operationally that becomes a big issue.” **(Participant 14, Executive Leader)** “So there could be some retailers that just don’t at all want to do anything that’s going to cost them more money, if it’s something that they feel isn’t 100% necessary, I guess, for them to do on their end of things.” **(Participant 4, Retail Associate)**
Implementation facilitators	“Yeah. I think that would actually probably even improve things, to be honest, because I do get a lot of people that come in and they’ll ask about safety classes. . .That could definitely help the business side of things for sure. . . it’s good incentive for them to come back and say, ‘Hey, I want to keep trying with these guys and do some more classes with them, ’ or something.” **(Participant 9, Range Officer and NRA-certified Instructor)** “That’s how you build rapport. That’s how you build an at-ease customer. It exudes and shows that you care about the community, you care about safety, you care about firearms, and you care about them. And I think that’s the real opportunity.” **(Participant 8, Lead Instructor and Range Director)** “Customers wanna feel like they’re actually not just getting a good product, but they’re getting what they came for, which is to feel safer.” **(Participant 4, Retail Associate)** “In all, in my experience, it only makes business better. You [the customer] know that I am concerned about you and the use of your firearm. . . I think it does a wonderful thing . . . They are now a lifelong customer. . . they will be back because they know we had their best interest at heart. Not just trying to take their money for the sale of a gun.” **(Participant 12, Director)**

### Theme 1: Firearm Retailers Find the Implementation of Point-of-Sale Firearm Safe Storage Programs Acceptable in the Retail and Range Setting With Proper Training

Nearly all retailers expressed support for implementing safe storage counseling at the point of sale. They commonly stated that they favored the concept, with one range officer saying, “I think that’s a really good program to instill for sure.” Another participant elaborated on why they found this program acceptable, stating:

I would love to have an opportunity to find out new flaws, these new understandings, get into the more mental psyche, learn more information about warning signs, being able to interact with people better and make [customers] feel more comfortable while being safe. Absolutely. (Participant 8, Instructor and Range Director)

While the overwhelming majority of retailers viewed the point-of-sale program favorably, it was evident that retailers needed proper training to ensure successful implementation. In particular, one participant stood out as an outlier and was not as supportive as the others. This participant believed the feasibility of carrying out the intervention was too great to realistically overcome. While the participant admitted to some merits of the proposed ideas, he or she ultimately concluded that it was impractical to pursue.

### Theme 2: Retailers Believe the Program Is Appropriate in the Retail Setting but Recognize It Might Be Received Differently by Certain Populations Over Others

Nearly all retailers believed implementing counseling at the point of sale was appropriate and relevant for promoting safe storage. However, some mentioned receptivity would likely differ between new and established gun owners. On the whole, retailers felt new purchasers would be more open to counseling compared to experienced owners who may already have ingrained storage habits.

Of those noting varying levels of appropriateness of intervention by customer attributes, several retailers still expressed value in providing point-of-sale counseling universally to all customers—including established owners who could benefit from updated information and reminders about safe storage practices. Overall, the majority of retailers agreed that point-of-sale firearm safe storage counseling programs were acceptable in the retail setting and had the potential to promote safer storage, especially among new firearm purchasers.

### Theme 3: Retailers Note the Need for a Balance Between Standardized and Adaptable Programs Given the Wide Variability in Gun Establishment Settings

In addition, several retailers emphasized the importance of developing point-of-sale programs that balance standardization with adaptability to accommodate variability across retail settings. They argue that consistent, standardized messaging and training are needed to ensure quality implementation.

However, prescriptive one-size-fits-all programs are unlikely to work, given differences in resources, layouts, staffing models, and organizational capacity across establishments. As such, retailers recommend that programs allow some level of customization to enable the delivery of point-of-sale anticipatory guidance with greater fidelity.

### Theme 4: Retailers Express a Full Spectrum of Self-Efficacy in Delivering Program Components

Retailers express differing levels of self-efficacy regarding their capability to deliver various facets of the proposed safety counseling intervention. For example, many participants convey high confidence in their skills to disseminate basic firearm safety information and discuss safe storage practices. However, several others noted low perceived self-efficacy in broaching the sensitive topic of suicide risk and prevention with customers. For instance, in responding to a question related to thoughts on how the program might impact different types of firearm-related injury, one retailer expresses a sense of fatalism surrounding suicide prevention:

Accidents, yes. Suicides? I’m not sure about it because it’s hard to prevent . . . I don’t know how you could try to prevent that other than seeking help on an individual level or telling people that you know who show signs of depression and they own firearms, getting them the help they need. (Participant 10, Retail Associate and Armorer)

In addition, other retailers mentioned that they were unaware of the signs and symptoms of mental health conditions and did not want to assume an individual’s mental health status:

There could definitely be an opportunity to provide the information out to the public or even to the retailers. Like I told you earlier, I’m not even personally aware of what all the specific signs are for depression. I might see a guy and say, “He looks kind of off. He looks like he’s, you know, just acting kind of weird.” (Participant 9, Range Officer and NRA Certified Instructor)

Some fell in the middle, indicating moderate self-efficacy and a belief that they could handle the counseling with sufficient training and ongoing support.

### Theme 5: Staffing Issues, Employee Consistency, Limited Time, and Costs Are Barriers to Program Implementation

Another major theme identified was implementation barriers which could be categorized into two types: time and cost burdens. Some retailers worried that extended transaction times from point-of-sale intervention might decrease the number of customers served, especially during busy hours, potentially turning away impatient customers.

Costs of extra staff training and any necessary technology were also raised as potential concerns for implementation. Beyond time limitations and financial costs, several participants noted issues in organizational capacity and employee consistency. More specifically, staff shortages and turnover could make training and proper execution of the intervention challenging.

### Theme 6: Leveraging Existing Firearm Safety Consultation Practices Within the Gun Establishment Facilitates Program Implementation

Finally, a few retailers described the distribution of safety information and safety training classes as already ingrained in their standard practices when selling firearms. In addition, they expressed confidence in their expertise in safety devices, particularly ones sold at their retail establishment:

The people that are buying safes, they’re gonna buy either the biometric safe, where it’s got the thumbprints on it, or the coded, where you enter in a digit code and you’re getting to your firearm fairly quickly. For the people that have larger collections that they have specific hunting rifles for specific hunting, so depending on what you’re hunting, you’ve got a different firearm. So, you have the big, stand-up safes, and that’s to keep those stored in the home when you’re not using them, because they’re only for hunting purposes. I think you’re gonna see less—and again, this is just from my knowledge at our store—you’ll probably see less of the trigger locks purchased. I’m not sure why. I know that each firearm, we provide a little lock, just the general lock that goes through the magazine and the slide-lock. (Participant 6, Retail Associate)

Many highlighted the belief that this program could improve store culture and cultivate a community. In the words of a director, counseling customers on firearm handling and firearm safe storage was seen as a rapport-building task that signaled to their customer base that “you care about the community, you care about safety, you care about firearms, and you care about them.”

Fostering point-of-sale conversations were also perceived as helping profits, as evidenced by a few retailers. Overall, this sample of retailers perceived safety counseling as core to their role and beneficial for both employee assurance and customer trust.

## Discussion

Altogether, these findings indicate general retailer support for incorporating firearm safety interventions into point-of-purchase interactions. Specifically, participants strongly favored the implementation of these programs into their workflows and provided key insights into target populations who might be more receptive to the intervention (e.g., new and first-time firearm owners). Against the backdrop of a firearm purchasing surge—in which first-time ownership is disproportionately increasing across purchasing segments (Schleimer et al., 2021)—findings highlight the promise of standardized programs leveraging point-of-sale relationships to effectively and responsibly reach firearm owners.

Although the intervention was rated favorably, participants did not receive all aspects equally. In particular, retailers were hesitant in having to identify customers struggling with depression and/or suicidality and lacked confidence in navigating conversations with patrons surrounding responsible firearm ownership while living with mental health challenges. These findings indicate that firearm safety interventions designed to be delivered at point-of-sale should equip retail staff with skills to recognize customers in mental health crises, support them, and navigate related conversations confidently. Successful implementation hinges on program planners thoroughly training staff to effectively conduct nuanced interpersonal counseling. This program could build upon the foundation of Mental Health First Aid (MHFA), a preventive program designed to teach community members to identify and assist those experiencing a mental health crisis. Meta-analysis of this program has shown that MHFA increases mental health literacy and improves support for those struggling with mental health, particularly within 6 months of training completion (Morgan et al., 2018). When designing initiatives, formal instruction alongside technical assistance resources should be explored to address retailers’ lower confidence when broaching sensitive topics like suicide risk.

Overall, four implementation barriers were identified for point-of-sale firearm safety programs in the retail setting: staffing issues, employee consistency, limited time, and related program costs. These barriers parallel important constructs from Proctor and colleagues (2011). Specifically, the financial costs and time constraints identified by retailers speak to the idea of implementation costs, whereas staffing issues and employee turnover relate to the idea of sustainability.

Encouragingly, several implementation facilitators also emerged from conversations with retailers. Participants suggested point-of-sale safety counseling is already somewhat incorporated into existing workflows and, thus, unlikely for implementation to disrupt operations. These findings differ from one 2002 investigation, where researchers found that when asked by customers, the majority of retailers did not offer information about the safe storage of firearms (Sanguino et al., 2002). However, there are important contextual distinctions between our respective studies. First, Sanguino and colleagues conducted their research in Chicago and Kansas City, where the firearm culture is likely very different from the one in our study. Furthermore, this research was published in 2002, which is over 20 years prior to when the present study was conducted. Since the early 2000s, there have been many cultural and political movements that have impacted the discussion surrounding gun safety (Zoller & Casteel, 2022). Second, in a study examining temporary firearm storage programing in gun establishments throughout Texas, findings published by McKay and colleagues (2022) determined that while firearm retailers ranged in their communication tactics, many retailers commonly discussed safe firearm storage techniques with their customers. Given these mixed findings stemming from particular geographic and cultural landscapes, future work should aim to investigate the degree to which conversations surrounding firearm safe storage are already occurring within the firearm retail and range environment across the nation.

Many voiced that reinforcing training opportunities would improve business and amplify profits, customer loyalty, and satisfaction. Furthermore, many retailers suggested that this customer–retailer interaction ultimately helped foster trust between agents in addition to serving as an opportunity to sell safety devices, including safes, locks, and other external safety devices. While the promotion of purchasing this equipment was not relayed to retailers during the interview, a theme emerged. It is a natural connection, as many retailers feel they have expertise in discussing safe firearm storage, and their knowledge is tailored to their store.

As the first in-depth investigation establishing proof of concept for point-of-sale firearm safe storage interventions deployed in gun establishments, this study makes a novel contribution. However, limitations must be acknowledged. First, the narrow sample from a single suburban store limits generalizability. Additional research should be replicated across more diverse establishments to determine consistency in staff perceptions. Recruiting through the workplace may have introduced social desirability bias among employees regarding employer-favored responses. Alternative enrollment approaches offsite could mitigate this concern. Finally, future directions should expand inquiry across other retail contexts like pawn shops, discrete ranges, and sporting goods stores to produce more composite understandings of implementation potential within this sphere.

Currently, evidence is severely limited, with only one study linking retailer discussions to subsequent safety behaviors (Bandel et al., 2023). While an initial step, this was a retrospective study, and observational data precludes determining intervention efficacy. Despite existing shop-based storage initiatives (Pierpoint et al., 2019; Polzer et al., 2021) structured protocols promoting customer conversations remain undeveloped and untested.

## Implications for Research

The themes from this project should primarily be applied in interventions for medium-sized community-centered retail locations. Future research should be conducted to explore these questions in other types of retail and range locations, i.e., small locally owned shops versus large commercial stores. Because this was an exploratory proof-of-concept study, we did not give the participants specific details about a proposed intervention in order to better gather broad perspectives and ideas for refining the intervention concept.

Future research surrounding evidence-based interventions within a retail setting should address the conclusions identified in this study. Concerns about resources (time and funding) could be mitigated in a few ways. Retailers could be given a script to expedite time. To address funding concerns, shareholders might consider applying to external grants and partnering with different academic and community groups. In recent years, several efforts have successfully demonstrated that public health and academic institutions can work collaboratively with firearm retailers to promote suicide prevention and secure storage efforts. For instance, Wright-Kelly et al. (2024) detail how Colorado’s Gun Shop Project has bolstered relationships between academic institutions and firearm businesses to adopt suicide prevention practices in retail settings (Wright-Kelly et al., 2024). In addition, a literature review from Henn and colleagues concludes that community-based firearm stakeholder-engaged interventions allow for further dissemination of safe firearm practices and increased suicide prevention (Henn et al., 2019). In particular, given recent changes to federal funding mechanisms that provide funding for community-engaged projects, there exists a ripe opportunity for academic-community partnerships in this space.

Future studies should investigate whether firearm establishments implementing point-of-sale firearm safety interventions may experience differential profits and sales of gun safety products compared to those not delivering interventions. In addition, trends in customer loyalty and levels of foot traffic with and without the program should also be tracked to garner meaningful insights in this space. Ultimately, results from these economic evaluations could yield persuasive evidence to alleviate possible reservations among prospective program adopters who may be anxious about the impact of the program on business operations.

Given the urgency for evidence-based firearm injury prevention, it is important to consider hybrid trials evaluating efficacy while gathering contextual factors influencing real-world implementation of point-of-sale interventions (Curran et al., 2012). Systematically developing and rigorously assessing standardized point-of-sale programs through experimental designs is critical. These efforts can elucidate efficacy and provide contextual insights to inform translation and dissemination across settings.

## Implications for Practice

Drawing from the findings of this study, we have constructed a comprehensive logic model for implementing point-of-sale firearm safety counseling interventions within retail environments (Figure 1). Broadly speaking, this model not only outlines the key components and activities of the intervention but also serves as a foundational framework and resource for scholars and practitioners to further design evidence-based strategies aimed at enhancing firearm safe storage practices among purchasers. In addition, this logic model can serve as a testable theory-of-change to evaluate similar firearm injury prevention programs and guide health promotion practitioners in their prevention efforts. By integrating these interventions directly into the retail and range setting, we aim to influence consumer behavior at a critical decision-making juncture. This model represents the initial step toward building a robust evidence base that can guide future efforts in injury prevention interventions.

**Figure 1:**
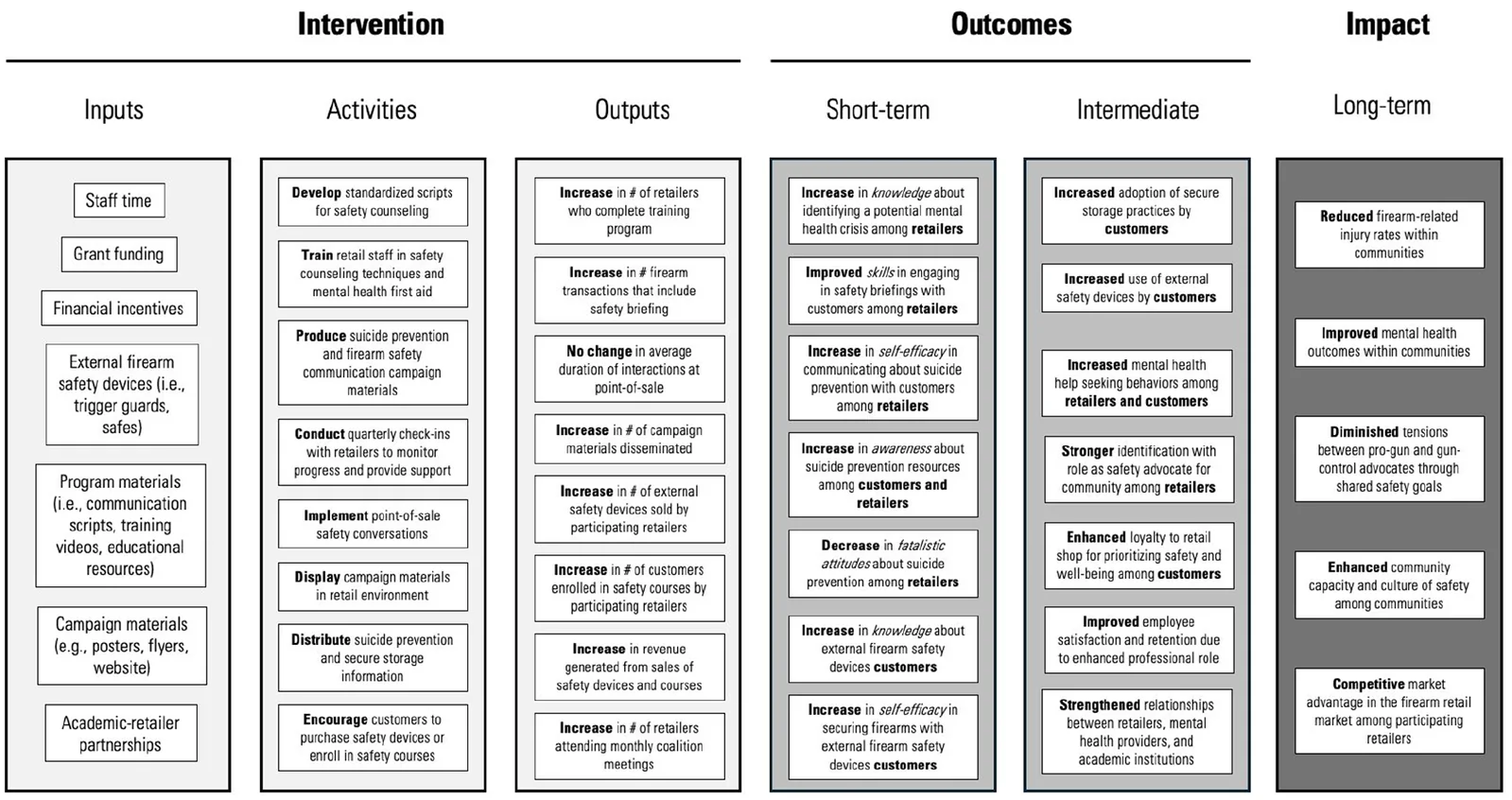
Proposed Logic Model for Firearm Secure Storage Counseling Intervention Delivered at Point-of-Sale in Firearm Retail Establishments

Powell and colleagues (2015) provide a comprehensive list of 73 strategies to address salient barriers to the implementation and dissemination of evidence-based interventions. Aligning with these recommendations and the results that surfaced from this analysis, there are several implementation strategies that might be beneficial to employ when considering embedding firearm safety anticipatory guidance programs in the retail and range setting. To mitigate costs, the focus could be on partnerships between retailers and academic and community groups to access funding streams supporting these firearm safety initiatives. In addition, lower-cost methods, such as posters, pamphlets, or other visuals, can be an effective communication tactic (Henson-Garcia et al., 2024). Using these tactics to passively communicate more sensitive topics, such as mental health and suicide, could simultaneously reduce expenses while allowing retailers to focus on things they feel more confident about, such as firearm handling and safe storage.

In alignment with the Diffusion of Innovations Theory (Dearing, 2009), we propose that such pioneering partnerships could serve as catalysts for the adoption and widespread dissemination of the intervention across broader networks. This concept is supported by research, such as the qualitative study by Polzner and colleagues (2020), which explored barriers and facilitators to community-driven suicide prevention partnerships between public health officials and firearm retailers. Their findings highlight the importance of engaging local gun groups, as these entities are trusted voices within their communities and can facilitate “entry into larger community engagements and sources of knowledge” (Polzer et al., 2021). In addition, program planners should consider establishing formal implementation blueprints to clearly outline protocols to ensure consistent program implementation across time and regardless of varying levels of staffing and employee turnover.

Overall, the favorable perspectives on appropriateness and supportive infrastructures signal that firearm retail establishments represent opportune intervention settings to ultimately mitigate firearm-related injuries that occur in communities across the nation.

## Conclusion

This qualitative study suggests that firearm retailers perceive point-of-sale programs as acceptable and appropriate for promoting safer storage practices among customers. Retail staff indicated high openness to integrating safety discussions into firearm sales transactions, especially for new and first-time buyers who represent a growing market segment of firearm owners. However, retailers noted that implementation would require formal training to bolster self-efficacy to deliver more sensitive components of counseling (i.e., mental health topics). Retailers cited some logistical barriers regarding staff capacity, time, and costs. They also highlighted established safety messaging practices and loyal customer relationships as assets facilitating smooth program assimilation.

## Supplemental Material

sj-docx-1-hpp-10.1177_15248399251323898 – Supplemental material for Establishing Proof-of-Concept for the Feasibility and Implementation of a Point-of-Sale Firearm Safety Counseling Program: A Qualitative Case Study Supplemental material, sj-docx-1-hpp-10.1177_15248399251323898 for Establishing Proof-of-Concept for the Feasibility and Implementation of a Point-of-Sale Firearm Safety Counseling Program: A Qualitative Case Study by Amanda Koong, Mike Henson-Garcia, Lauren Malthaner, Katelyn Jetelina, Belinda Hernandez and Sandra McKay in Health Promotion Practice
