# Supplementary material for: Establishing Proof-of-Concept for the Feasibility and Implementation of a Point-of-Sale Firearm Safety Counseling Program: A Qualitative Case Study
Source: Health Promot Pract. 2025 Mar 24;27(2):285–96. doi: 10.1177/15248399251323898 (PMC13687624; doi:10.1177/15248399251323898)
Supplement: Supplementary file 1 [file sj-docx-1-hpp-10.1177_15248399251323898.docx]

**Employee Team Member – Interview Guide**

| **Topic** | **Interview Questions and Probes** |
| --- | --- |
| **Getting Started** | “Welcome and good (morning/afternoon/evening). My name is _______, and I am a Ph.D. student from the ____________ working on research project on firearm safety.  Overall, our goal is to hear from firearm retailers like yourself on firearm safety programs. We have some general questions about your personal opinion related to these matters. For your participation, we will be providing you a gift card in the amount of $50.  All of the information you provide will remain strictly confidential. None of our notes or summaries will have any identifying information about you. We will never identify an individual by name or location. But, if there is a question that you don’t want to answer, we can absolutely skip that question or stop the interview.  We anticipate that the interview will last no longer than an hour. We are also hoping to record this session, as it will allow me to focus on our conversation rather than furiously notetaking.  Are you willing to participate in this interview?  [If yes, start the interview. If not, “Thank you for your time.”] |
| Current job duties | - Could you briefly tell me about your current job and what it entails?   - Probe: How often do you interact with customers on a daily basis?   - Probe: How long have you been with your current employer? - Could you walk me through a typical interaction with a customer?   - Probe: Could you walk me through your thought process on making sales in your store? - Thinking a little bit more about your interactions with customers, do you ever provide firearm safety information?   - IF NOTHING: Do you think there is a need for providing this information to customers? If so, what information should be given out? - What type of firearm safety information do you give out to customers?   - Probe: Who are the sources or authors of the information you give out?   - Probe: Do you think the materials are attractive or persuasive? Why or why not?   - Probe: How do you think this method of giving out information compares to other options (like word-of-mouth, posters, websites, or phone apps)?   - Probe: Do you provide information related to suicide prevention? What about information related to accident prevention?   - Probe: Do you think there is a need for providing this information to customers? - Does the type of customer (appearance, gender, age, etc.) influence the type of information you provide on safety? |
| Knowledge of firearm safety practices and devices | - In your opinion, what are the most effective safety devices that exist on the market? - Do you think that the experience of the firearm owner matters in the type of safety device that you would recommend to them? - What safety devices are popular among your customers? |
| Attitudes toward firearm safety | - Could you tell me your thoughts, opinions, and feelings about firearm safety?   - Probe: Do you think firearm safety is an important issue? Why or why not?   - Probe: Are there issues that are more important than firearm safety? If so, why and what are they?   - Probe: Do you think the way we commonly think about firearm safety needs to change? If so, how? |
| **Segue** | **This next series of questions are going to be related to a proposed safety program. We are interested in your opinions about whether it’s feasible in your work environment. Our idea is that employers like yourself would provide firearm safety information and counseling at the point-of-sale. When we talk about providing information, we generally mean the provision of firearm safety guidance through means other than conversation – such as through pamphlets, flyers, posters, advertisements, mobile apps, and websites. On the other hand, when we talk about counseling, we generally mean the provision of firearm safety guidance via one-on-one conversations with customers at the point-of-sale.**  **In our program, we would train firearm retailers to provide information and counsel individuals around three different topics: (1) proper firearm storage, (2) keeping firearms away from children, and (3) keeping firearms away from those experiencing suicidal ideation. Keep this information in mind as we go through the next series of questions.** |
| General concept testing | - What are your general thoughts about the program concept we just described?   - Probe: What are the components that would work well in the retail/range environment?   - Probe: What are the components that wouldn’t work so well in the retail/range environment? - Do you feel like the right person to be providing counseling like this to firearm customers? Why or why not?   - Probe: Who do you think should be providing information like this? - From a business perspective, what would be the important factors to consider when integrating firearm safety counseling at the point of sale?   - Probe: How might the integration of firearm safety counseling at point-of-sale interfere with business if at all? |
| Perceived self-efficacy in providing intervention | - Could you describe how confident you feel in providing the intervention at this very moment?   - Probe: What would help you feel more confident to do something like this if anything? |
| Anticipated resources and costs for implementation | - What might your organization need to start providing firearm safety counseling at point of purchase?   - Probe: Are there resources that your organization would need to do a program like this? If so, could you tell me what these would be? - Are there resources that you or your organization currently possess that would make this program a success to your organization? If so, what are these? |
| Perceived attitudes of intervention from customers | - What do you think your customers would think about firearm safety counseling at the point-of-sale?   - In other words, how do you think your customers would feel about being given firearm safety information during their transaction? - Do you think your customer base would be receptive to firearm safety information? Why or why not? |
| Perceived benefits and barriers of intervention | - What are some potential benefits of firearm safety counseling at the point-of-sale? - On the flip side, what are some barriers or challenges you can think of related to firearm safety counseling at the point-of-sale? - Are there any words or phrases you feel would be confusing or potentially offensive to customers when receiving firearm safety counseling? - How do these benefits compare to the barriers you just mentioned? - Do you think it will be challenging to integrate firearm safety counseling into your job duties or the duties of employees at your store? Why or why not? |
| Perceived effectiveness of intervention | - What impact, if any, do you think this idea has in your community? - Do you think firearm safety counseling would be an effective strategy in preventing firearm-related accidents? What about in preventing suicides? What about in preventing crime? |
| Perceived sustainability of intervention | - Do you think providing firearm safety counseling at the point-of-sale is a sustainable effort? Why or why not?   - Probe: What, in your opinion, are the important things that are needed to ensure an effort like this would be maintained over time?   - Probe: Do you think this program, if implemented, would be able to keep running for a long period of time? |
| **Open-ended and closing** | - That’s all the questions we have. Do you have any final thoughts about any of these topics or ideas? - **Thank you so much for your time. Again, as a thank you, we will be sending you a 50$ electronic gift card for your time today.** |
